# Developing a comprehensive, culturally sensitive conceptual framework of health domains in Singapore

**DOI:** 10.1371/journal.pone.0199881

**Published:** 2018-06-28

**Authors:** Julian Thumboo, Mandy Y. L. Ow, Elenore Judy B. Uy, Xiaohui Xin, Zi Ying Clarice Chan, Sharon C. Sung, Dianne Carrol Bautista, Yin Bun Cheung

**Affiliations:** 1 Department of Rheumatology & Immunology, Singapore General Hospital, Singapore, Singapore; 2 Office of Clinical, Academic & Faculty Affairs, Duke-NUS Medical School, Singapore, Singapore; 3 Yong Loo Lin School of Medicine, National University of Singapore, Singapore, Singapore; 4 Academic Medicine Research Institute, Duke-NUS Medical School, Singapore, Singapore; 5 Academic Clinical Programme for Medicine, Singapore General Hospital, Singapore, Singapore; 6 Office of Clinical Sciences, Duke-NUS Medical School, Singapore, Singapore; 7 Department of Child and Adolescent Psychiatry, Institute of Mental Health, Singapore, Singapore; 8 Singapore Clinical Research Institute, Singapore, Singapore; 9 Centre for Quantitative Medicine, Duke-NUS Medical School, Singapore, Singapore; 10 Tampere Center for Child Health Research, University of Tampere and Tampere University Hospital, Tampere, Finland; Janssen Research and Development, UNITED STATES

## Abstract

The increasing focus of healthcare systems worldwide on long-term care highlights the need for culturally sensitive Health-Related Quality of Life instruments to accurately capture perceived health of various populations. Such instruments require a contextualized conceptual framework of health domains, which is lacking in some socio-cultural contexts. We developed a comprehensive and culturally sensitive conceptual framework of health domains relevant to the Singaporean population. We recruited Singaporeans/ permanent residents, English/ Chinese-speaking, with/ without chronic illnesses to participate in focus group discussions (FGDs) and in-depth interviews (IDIs). We elicited health areas participants perceived to be important for them to be happy and satisfied with life. To encourage spontaneous emergence of themes, we did not specify any aspect beyond the broad domains of Physical, Mental, and Social health so as not to limit the emergence of new themes. Themes from the transcripts were distilled through open coding (two independent coders), then classified into more abstract domains (each transcript coded independently by two coders from a pool of six coders). From October 2013 to August 2014, 121 members of the general public participated in 18 FGDs and 13 IDIs (44.6% males, mean age: 53.3 years 77% Chinese, 9% Malay, 12% Indian, 63% with chronic illness) while 13 healthcare workers participated as patient-proxies in three FGDs. Thematic analysis identified 27 domains. The 15 physical domains included physical appearance, energy, physical fitness, and health and resistance to illness. The nine mental domains included emotions, self-esteem, and personal freedom. The three social domains were social contact, social relationships, and social roles. This conceptual framework reflected physical, mental, and social dimensions of well-being, suggesting that the Singapore population’s views on health support the World Health Organization’s definition of health as “a state of complete physical, mental and social well-being and not merely the absence of disease or infirmity”.

## Introduction

The increasing focus of healthcare systems around the world on long term care brings with it a need for holistic outcome measures for health. In this regard, Health Related Quality of Life (HRQOL) is an important outcome measure because it evaluates a patient’s perception of his or her health [1], and complements the traditionally used measures of morbidity and mortality. Importantly, HRQOL reflects an individual’s degree of health as opposed to the presence or absence of disease, and thus aligns with the WHO definition of health as a "state of complete physical, mental, and social wellbeing, and not merely the absence of disease or infirmity" [2].

Measuring HRQOL is especially important for the management of chronic diseases, which are generally not immediately life-threatening and do not have a cure. Further, chronic diseases often affect multiple domains of the health of their sufferers, including physical, psychological and social functioning, which cannot be fully assessed through traditional methods used to assess morbidity and mortality [3]. However, most existing HRQOL instruments have been developed in the West, and intended for use in Western cultural contexts, and have thus been based on Western conceptions of health. There is clear evidence that perceptions of health and illness differ in different socio-cultural contexts. For example, in the United States, illness is conceptualized in terms of severity and contagiousness, while in Singapore, there is an additional dimension of spiritual/ psychological causation [4]. Several HRQOL instruments (for example the SF-36 and EQ-5D) have been adapted for use in other socio-cultural contexts, including Asia. Our previous work to adapt selected western instruments for use in Asian contexts has highlighted two main issues. First, there is difficulty in fully adapting these instruments for use in an Asian context [5], partly because of the lack of an underlying framework of health domains. Second, the different conceptualizations of health in western versus Asian socio-cultural contexts can affect their validity and usefulness of some HRQOL instruments in measuring HRQOL. For example, the “role emotional” domain of the SF-36 measures physical more than mental components of health in several Asian countries [5, 6].

This study aims to address these issues by establishing a comprehensive and culturally sensitive conceptual framework of health domains based on input from individuals in the Singapore population. The long-term goal for the development of a culturally sensitive framework of health for Singaporeans is to incorporate this measurement of HRQOL into routine clinical practice to improve the quality of life of patients with chronic diseases. As this framework is based on input from local participants’ perspectives, it will have high content validity. The perspectives of the individual patients and healthy individuals will also represent a broad spectrum of Singaporean population, thus increasing the external validity of this framework. This high content and external validity would allow this conceptual framework of health to be a useful reference for the construction of individual and population health profiles in our society. This robust framework is also the foundation for the development of good empirical measurements of health in Singapore, and by extension, potentially in other Asian socio-cultural contexts.

## Methods

### Participant recruitment

Consenting adult members of the general public and healthcare professionals (HCP) participated in focus group discussions (FGDs) or in-depth interviews that were conducted from October 2013 to August 2014. This study was approved by the SingHealth Centralised Intuitional Review Board (ref2013/502/A).

To ensure representativeness of the views of the adult (age≥ 21 years) Singaporean population, a purposive sample was drawn based on age, gender, ethnicity, education, and presence or absence of 10 most commonly reported chronic illnesses in Singapore based on the 2010 Singapore Burden of Disease Study [7]. These 10 conditions were diabetes, heart disease, lung/ breast/colon cancer, osteoarthritis, stroke, asthma or chronic obstructive pulmonary disease (COPD), anxiety, depression, migraine, vision disorders, or adult-onset hearing loss. Individuals with impairments that precluded their participation in a meaningful exchange of ideas (i.e. severe mental illness, cognitive impairment or other conditions that prohibited them from carrying out a normal interview) were excluded from the study.

Potential study participants were recruited though posters and multi-media advertisements, which were displayed in waiting areas of public healthcare institutions including primary care outpatient clinics, hospital based specialist out-patient clinics (SOCs) and a community center, and through word of mouth. Targeted recruitment of participants with chronic conditions was carried out in selected SOCs in the Singapore General Hospital (SGH), the Singapore National Eye Center (SNEC), or the National Heart Center (NHC), through referral by their attending physician or nurse clinician identification through a review of case notes and electronic medical records.

### Focus group discussion and individual interviews

Focus groups and individual interviews were conducted in English or Chinese (Mandarin or Cantonese) in a quiet room in the same campus as the recruitment site, and lasted approximately 60 to 120 minutes. Each focus group consisted of two to eight participants. Only participants and members of the study team were present during the session. To ensure that each FGD was attended by a mix of participants, we reviewed the list of potential participants and purposively sampled FGD participants based on their availability, and individual characteristics (i.e. age, gender, ethnicity, presence or absence of any chronic conditions, and educational background). Although participants may find meeting others with similar interests or similar levels of understanding of a given topic more appealing than meeting with those who are perceived to be different [8], we included a mix of participants because a homogeneous group of healthy participants may find it difficult to have any in-depth discussion on health. In contrast, participants with illness who have direct experiences of what it is like when they are not in optimal health would find it easier to pinpoint specific domains and items that are most important for them. The domains and items offered by these participants can provide a useful context to help healthy participants to reflect on and share their health experiences, which may complement the experiences of the ill participants.

All focus groups were facilitated by a moderator; another member of the team served as an observer and a note taker. In total, six moderators/interviewers conducted all focus groups/interviews. Moderators/ interviewers were female (n = 6), with Bachelors (n = 1), Masters (n = 3) or PhD (n = 2) training in sociology (n = 1), qualitative research (n = 4), and psychology (n = 1). Six facilitators were working in research in our academic medical center and were working full time (n = 2) or part time (n = 4) on this project. The facilitators had no relationship with the study participants prior to study commencement. During session introductions, interviewers shared only their name, job title and role in the project with study participants.

Moderators/interviewers used a standardized guide (see supplementary materials) developed in accordance with a phenomenological approach. This approach is used to study lived experience and questions were designed to be open-ended to minimize the influence of interviewer probes on participant responses [9].The draft guide was refined after two pilot focus groups. In part one of each FGD session, participants were asked to complete an individual exercise in which they thought of someone, about their age, who was happy and satisfied with life as a whole. Participants were asked to list the areas of life they considered important in order to be happy and satisfied with life. Participants were then asked to discuss these areas in the group discussion. In part two of the session, participants were asked to complete a similar exercise, but to only focus on health and discuss the subset of areas in health. In part two, the standardized guide included probes for three broad domains of health—physical, mental and social health–to ensure that these broad areas were covered if not spontaneously brought up by participants. One FGD session was conducted in Mandarin to determine if there were any new themes that would emerge among older-generation, Chinese-speaking Singaporeans. Fieldnotes were not taken during FGD and individual interviews.

We conducted individual interviews for a select group of patients who would not otherwise be able participate in a FGD, using the same standardised moderator guide and exercises. This included patients with impaired mobility, requiring extensive assistance with activities of daily living (ADL), or moderate to severe hearing loss or speech slurring. Towards the later part of the study, patients who were part of a pre-specified group of interest, for whom recruitment fell below the targetted numbers, and were unable to commit to attending a future FGD, were also recruited to participate in individual interviews. In both these instances, the decision to allow for individual interviews was made to better ensure representation of these subsets of patients who would have othewise not attended an FGD.

We conducted FGDs with healthcare-professional participants, who served as patient proxies, to obtain additional perspectives on the domains of health of importance to patients in Singapore. These groups were conducted in accordance with the methods used in the development of the World Health Organization Quality of Life (WHOQOL) assessment instrument [10]. We purposively sampled healthcare professionals to provide broad representation of the various healthcare professions (doctors, nurses and nurse clinicians, physiotherapists, occupational therapist, radiation therapist, pharmacist, social workers, and standardized patients). The standardized guide was similar to the above but instead asked health care professionals to think about patients whom they work with, and consider to be happy and satisfied with life as a whole.

### Data analysis

Thematic analysis of the qualitative data was carried out using NVivo 10 software (QSR International Pty Ltd, 2012).

Focus groups and interviews were voice-recorded and transcribed verbatim. Themes were distilled from transcripts using thematic analysis. Interviews conducted in Chinese were transcribed in Chinese and subsequently translated to English; analysts referred back to the verbatim Chinese transcript and/or the voice recordings as necessary. An initial codebook was developed by independent open coding of transcripts. Open coding is a data-driven process to identify emerging themes directly from data, rather than using ideas from existing literature or frameworks [11]. Thirty-four transcripts (21 FGs, 13 individual interviews) were coded by six analysts as follows: MO and CC performed open-coding on five transcripts and achieved consensus to develop the initial version of the codebook. To ensure that codes in the codebook could be consistently applied, SS and SM independently coded two additional transcripts using the initial codebook over two more coding rounds. These four analysts held a consensus meeting after each coding round and iteratively refined the codebook, resulting in a revised codebook. MO, an analyst involved in the creation of the codebook from its inception and EU, an analyst not involved in coding any of the previous transcripts, independently coded five transcripts using this revised codebook and found consistent application of the codebook between coders. This codebook was used to code subsequent transcripts, during this process, new subthemes were added but no new themes were identified. After the 3rd panel meeting (detailed below), three analysts (CC, EU, and XX) coded transcripts from two focus groups and 11 individual interviews. New codes were added to the framework after coding one additional FG and three individual interviews. No additional codes were generated from the analysis of one additional FG and six individual interviews. At this point, the study team reached consensus that no additional concepts were identified, and thus qualitative data saturation had been reached.

Three expert panel meetings were convened after the analysis of the 4th, 16th, and 19th focus group transcripts to provide additional input on the evolving framework. Each expert panel was comprised of members of the study team and external experts. After each expert panel meeting, we created new codes to encompass the themes in the post-panel review framework. The revised set of codes was then used to analyze the subsequent transcripts.

The final output of the thematic analysis, with input from expert panel meetings, was a list of descriptive themes of HRQOL domains and their related sub-themes deemed important to the Singapore general population, based on the discussion and elaboration of our participants. The definition of each domain was derived from transcripts through an iterative process of discussion involving the study team with review by the expert panel. We developed both comprehensive and simplified domain definitions, the former for use by researchers, and the latter for use by participants in future studies. The placing of a domain in each area followed a similar iterative process of discussion with the study team and review by the expert panel.

## Results

### Demographics

Of the 365 individuals who indicated interest to participate in the study, 292 preferred to participate in an English session and 73 preferred Mandarin. Five of these individuals were found to be ineligible to participate due to a limited ability to communicate in English or Mandarin (i.e. participant spoke a Chinese dialect, n = 2), significant hearing impairment (n = 1), dysphasia (n = 1), or age <21 years old, while other participants, though eligible, did not participate because of scheduling issues or did not attend their scheduled sessions. A total of 134 individuals participated in 21 FGDs and 13 individual interviews, which were conducted over a 10-month period, from October 2013 to August 2014. There were 18 general population FGDs (15 conducted in English and three in Mandarin) and three health care professional FGDs (all conducted in English). Each of the 21 FGs was comprised of two to eight participants, with majority of the focus groups having six to seven participants (11 FGs). The 18 general population FGDs ranged in size from four to eight subjects, with 14 to 100% of participants female and three, 14 and one sessions conducted in the morning, afternoon and evening respectively. The three healthcare professional FGDs ranged in size from two to seven subjects, with 71 to 100% of participants female and one and two sessions conducted in the afternoon and evening respectively.

Saturation of the topics occurred after twenty sessions, with no new themes emerging after the twenty-first session. The demographic details of FGD and in-depth interview participants are summarized in Table 1.

**Table 1:** Demographic profile of study participants and the 2014 resident population in Singapore [12].

Frequency (%)	Focus group discussion participants (%) n = 121	Individual interview participants (%) n = 13	Singapore resident population 2014 (%) n = 3,870 (‘000)
**Age**			
Median	54	56	39.3
Min	22	41	0
Max	85	80	N>100
**Age (range)**			
<35	23 (19)	0 (0)	1,677 (43)
35–54	40 (33)	6 (46)	1,242 (32)
≥ 55	58 (48)	7 (54)	951 (24)
**Ethnicity**	** **	** **	
Chinese	96 (79)	8 (62)	2,874 (74)
Malay	6 (5)	5 (38)	516 (13)
Indian	17 (14)	0 (0)	353.0 (9)
Others	2 (2)	0 (0)	126.7 (3)
**Gender**			
Male	59 (49)	5 (38)	1,902.4 (49)
Female	62 (51)	8 (62)	1,968.3 (50)
**Years (level) of education**^‡^			
0 to 6 years (primary)	9 (7)	2 (15)	833.3 (31)
7 to 12 years (secondary)	52 (43)	7 (54)	733.6 (27)
≥ 13 years (tertiary)	59 (49)	4 (31)	1102.3 (41)
Undeclared*	1 (1)	0 (0)	—
**Self-Report of at least one top 10 disease conditions**^†^			
Yes	41 (34)	1 (8)	Not available
No	65 (54)	12 (92)	Not available
Undeclared*	15 (12)	0 (0)	Not available

*Subject chose not to provide this information

^†^Based on Singapore Burden of Disease Study [4, 5]

^**‡**^Percentages are for 2013 population data

### Framework for health domains

**Fig 1:**
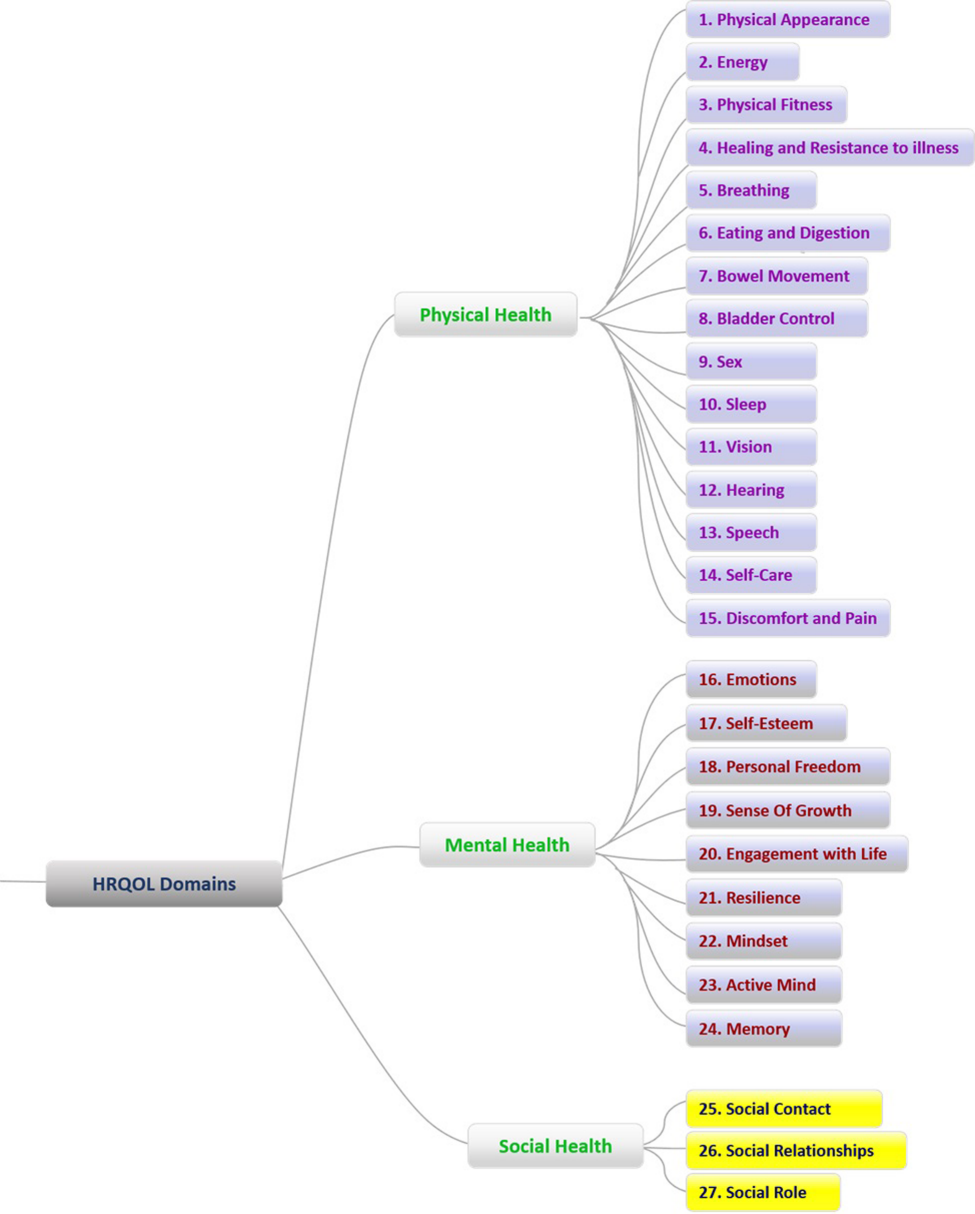
Health domains framework [10] with three components (physical, mental, social health) and 27 domains, one of the first in an Asian sociocultural context [13–15].

The framework for health domains resulting from this study is shown in Fig 1. Physical, Mental and Social Health were identified from data analysis as major components, with 15, 9, and three domains respectively, with no prior framework being used to pre-define domains of health during the focus groups or interviews. The definition of each domain and illustrative quotes are summarized in Table 2. Physical Health had the largest number of domains which broadly spanned the various aspects of physical health while encompassing several distinct groupings. Bodily functions were represented by the domains of breathing, eating and digestion, bowel movement, bladder control, and sleep. Of the five senses, vision and hearing were identified as domains. Energy, physical fitness and healing and resistance to illness formed another distinct grouping.

**Table 2:** Domain definitions and sample quotes [16].

No.	Domain	Operational definition	Simplified domain definition	Sample quote/s
1	Physical appearance	A person’s perception of his body in the physical sense: his weight, body size, or figure; appearing healthy; appearing youthful; not having any physical deformities.	Having normal physical appearance (acceptable weight, looking healthy).	“Have a moderate figure, not too fat, not too skinny.”*(FGD*: *44 years old*, *Chinese*, *female*, *healthy)* “Healthy looking. That means the appearance look good, look healthy.”*(FGD*: *61 years old*, *Chinese*, *female*, *healthy)*
2	Energy	A person’s energy to do physical tasks (i.e. level of lethargy or fatigue).	Having energy to do things.	“Actually my energy level is rather low, someone commented, “if you go out one day, you must rest two days”.*(FGD*: *68 years old*, *Chinese*, *female*, *with multiple medical conditions)* “They feel very lethargic. They don't have the energy to do the things that they want to do.”*(HCW*, *Registered Nurse)*
3	Physical fitness	A person’s capacity to do physical tasks with strength, agility, and stamina, and to move about without assistance from other people.	Being able to carry out physical activities and move around without difficulty.	“I can do brisk walking. Do the daily activities… you know, take up some sports, all these [sic]. Then, it should be ok.” *(FGD*: *56 years old*, *Indian*, *male*, *healthy)* “Able to move about independently. There’s no need to get any family member to accompany you each time you go out.”*(FGD*: *71 years old*, *Chinese*, *male*, *with multiple medical conditions)*
4	Healing and resistance to illness	A person’s ability to resist falling sick and to quickly recover from illness or injury.	Not falling sick easily and getting well quickly when I am sick.	“You’re fortunate enough, still strong in immunity. So whether it’s recovering from an illness…or maybe if you would have some sort of injury, maybe minor ones-scratches on arms or any scratches, cuts or burns, you probably [sic] be able to recover from it? Heal or topple it?” *(FGD*: *24 years old*, *Chinese*, *female*, *with multiple medical conditions*.*)* “I think your immune system is able to fight off all those virus [sic] is also important so you get sick very rarely. So you can optimise your performance in things that you do.”*(FGD*: *22 years old*, *Chinese*, *male*, *with multiple medical conditions)*
5	Breathing	A person’s subjective perception of the quality of his breathing (e.g. “being breathless” or “short of breath”).	Being able to breathe well.	“Now you want to do a little and short of breath already [sic].”*(FGD*: *66 years old*, *Chinese*, *female*, *with multiple medical conditions)* “So I just pray that every day I don’t feel breathless.”*(FGD*: *19 years old*, *Chinese*, *female*, *with multiple medical conditions)*
6	Eating and digestion	A person’s ability to eat (i.e. have a good appetite for food, chew and swallow well) and digest food (i.e. able to retain what has been eated, not having indigestion).	Being able to eat and digest food well.	“Being fed through a feeding tube. I have seen stroke patients. My dad was fed that way towards the end of his life. I felt (there was) no quality of life, and there is no meaning to it. He enjoyed food when he was young, that’s the worst part.” *(FGD*: *56 years old*, *Chinese*, *female*, *with multiple medical conditions)* “When you lie down the acidic [sic] will start to digest the food and you start coming forth your mouth and you {choking sound}.”*(FGD*: *52 years old*, *Chinese*, *male*, *with multiple medical conditions)*
7	Bowel movement	A person’s ability to move his bowels (i.e. not constipated).	Being able to pass motion regularly (not having constipation).	“… so she give me advice, tell me not to depend on medicine, how to overcome constipation all that and it works for me.”*(FGD*: *68 years old*, *Chinese*, *female*, *with multiple medical conditions)*
8	Bladder control	A person’s ability to control when and how frequently he empties his bladder.	Being able to control your urine.	“They [are] shy. . .then they don’t dare to tell us that they started to lose control of their bladder.”*(HCW*, *Radiation Therapist)* “But now, if I were to say, if I don’t wake up two to three times in the whole night is considered very fortunate already. Nowadays at night must frequently wake up to urine [sic].”*(IDI*: *59 years old*, *Chinese*, *male*, *with multiple medical conditions)*
9	Sex	A person’s satisfaction with his/her ability to engage in sexual relations.	Having a satisfying sex life.	“Having a good happy hour (sex) life with wife is also important.” *(FGD*: *49 years old*, *Chinese*, *male*, *with multiple medical conditions)* “And then this is…for example the husband have some sexual dysfunctioning [sic], then actually the marital relationship get worse. But somehow this (is) something taboo that may not really want to tell you.” *(HCW*, *Medical Social Worker)*
10	Sleep	A person’s ability to fall into and remain asleep in order to wake up feeling rested.	Being able to sleep well.	“Able to sleep well, no insomnia. I have lot of friends who couldn’t sleep well; some are still awake at two, three A.M. “*(FGD*: *45 years old*, *Chinese*, *male*, *with multiple medical conditions)* “And it will be very beautiful to eat and sleep well. “*(FGD*: *68 years old*, *Chinese*, *female*, *with multiple medical conditions)*
11	Vision	A person’s ability to see clearly (with or without glasses).	Having good eyesight (with or without glasses).	“I feel that being healthy means being able to see and hear clearly.”*(FGD*: *36 years old*, *Chinese*, *male*, *medical condition/s)*
12	Hearing	A person’s ability to perceive sound clearly (with or without a hearing aid).	Being able to hear well.	“Last time to me hearing not important. Now I got hearing problem, I already know it is very important. So now I must not only carry a hearing aid but also the battery… Even at home ah, sometimes it is so bad, I need to wear hearing aids. Even the phone ring, I can't hear. The doorbell ring, I can't hear.”*(IDI*: *59 years old*, *Chinese*, *female*, *medical condition/s)*
13	Speech	A person’s ability to speak clearly and be understood by others (e.g. no slurring of speech; clear pronunciation of words).	Being able to speak clearly so that others can understand (e.g. no slurring of speech).	“My speech is a bit blur…blur as in slurring. But I want to come back to work…and speak as clearly [sic] like last time.”*(IDI*: *50 years old*, *Malay*, *male*, *with medical condition/s)*
14	Self-care	A person’s ability to perform his own activities of daily living (ADL), i.e. feeding, bathing, grooming, toileting.	Being able to take care of self-care needs without help from others (i.e. eating, bathing, getting dressed).	“The most important I think is must be able to take care of your own needs, eating, bathing, walking, running. *(FGD*: *68 years old*, *Chinese*, *female*, *with medical condition/s)* “If you got health, usually you’d be able to take care of yourself.” *(FGD*: *50 years old*, *Chinese*, *female*, *healthy)*
15	Discomfort and pain	A person’s experience of bodily aches, discomfort, or pain—these can either be localised to specific parts of the body or involve the entire body (e.g. headache, joint pain, back pain).	No aches or pains in the body.	“Something that is wishful thinking at this point of time for me is to be free of aches and pains, because I do have [pain]. I am not [pain] free. But I can make use of balms and whatever.” *(FGD*: *68 years old*, *Chinese*, *female*, *with medical condition/s)* “Then when they are in pain, they cannot do the things they want, it makes them very miserable.”*(HCW*, *Registered Nurse)*
16	Emotions	The range of feelings that a person experiences; this domain centers on maximising positive feelings (e.g. happiness, joy) and minimising negative feelings (e.g. sadness, worry).	Being happy, not sad, angry, worried.	“I’m very happy. I always make myself happy.”*(FGD*: *61 years old*, *Chinese*, *female*, *healthy)* “So I try not to get so angry, so agitated. I try to cool down myself.” *(IDI*: *48 years old*, *Chinese*, *female*, *with medical condition/s)*
17	Self-esteem	A person’s confidence in himself and his abilities.	Having confidence in yourself	“Because you are deteriorating physically, you know that you are not able to do as much already so you start to have this sense of self-doubt. So when people need you, it adds on to your self-worth.” *(FGD*: *52 years old*, *Chinese*, *female*, *with medical condition/s)* “So it’s self-worth because when you lose your vision, you lose your employment, you lose your independence, this a normal onset [sic]. So what am I, at the end of day? Am I a burden to my family? So your self-worth somehow just plunges. The self-confidence also.”*(HCW*, *Nurse Educator)*
18	Personal freedom	His ability to do what he wants to do, without feeling that he is restricted by others.	Being independent and in control of your life.	“Meaning to say that I really can go anywhere in the world (that) I want to go without any restriction, do whatever that I want to do [sic]. To me, that’s the most important thing.”*(FGD*: *55 years old*, *Chinese*, *male*, *with medical condition/s)* “So I think that if I want my own freedom, everybody (else would) also want their own freedom, their own area of, you know, their own area of freedom.” *(FGD*: *61 years old*, *Chinese*, *female*, *healthy)*
19	Sense of growth	His ability to grow and mature as a person (i.e. knowledge, skills, and emotions).	Being able to grow as a person (i.e. knowledge, skills, emotions).	“So I get information from all sources and I think this also adds on to my sense of happiness. That I’m learning new things along the way in life.” *(FGD*: *52 years old*, *Chinese*, *female*, *with medical condition/s)* “I think what is important is that you don’t live a day that feels like it is a repeat of yesterday. To me whatever elements of my life needs to have a sense of progress, and yeah, not regression.” *(FGD*: *29 years old*, *Chinese*, *male*, *healthy)*
20	Engagement with life	A person’s ability to derive meaning from the activities he engages in.	Being able to find satisfaction and meaning in the things that you do.	“But I really find something to occupy myself. Like volunteer in some organisations, or like have their own interests, hobbies, or some of them like to travel. So I feel like for the elderly, for those retired, it’s good for them to explore. *(HCW*, *Nurse Educator)*
21	Resilience	A person’s ability to cope with and overcome life’s challenges.	Being able to overcome difficulties in life.	“We must not have any problems that overcome us. We must overcome the problem.”(*FGD*: *demographic details unavailable*) “Yeah, ups and downs sure have one lah [sic]. But through friends, they have taught me to persevere. Learn to persevere. Perseverance is very important, because when you can persevere, then I am sure you can carry on.”*(IDI*: *59 years old*, *Chinese*, *female*, *with medical condition/s)*
22	Mindset	A person’s attitude to life-cultivate being positive, avoid being negative	Thinking positively in life.	“So always stay positive I think (it is) very important. Don’t look at (things) too negatively.” *(IDI*: *demographic details unavailable)* “And also the person, himself or herself with a positive attitude or optimistic attitude tends to look at things in the brighter side, you know, ah ya [sic]. *(HCW*, *Registered Nurse)*
23	Active mind	A person’s ability to keep his mind engaged; to have a mind that is active and alert.	Being mentally alert and active (e.g. reading, learning new things, taking up a new hobby).	“Basically we have to keep our brain alert.” *(FGD*: *demographic details unavailable)* “Keep your mind active. Reading newspaper, whatever you read is ok. *(FGD*: *71 years old*, *Indian*, *male*, *with medical condition/s)*
24	Memory	A person’s ability to remember objects, thoughts, and past events (i.e. not forgetful).	Having a good memory (able to remember objects, thoughts, and events)	“Just that she always have [sic] to remind me…like I always forget thing [sic]. Even my go take bus, I also take the wrong bus [sic].” *(FGD*: *55 years old*, *Chinese*, *female*, *with medical condition/s)* “My children will help me remember because I will forget sometime [sic]. My children will take my medicine to me and remind me to take.” *(IDI*: *80 years old*, *Chinese*, *female*, *with medical condition/s)*
25	Social contact	The number of social interactions a person has with others.	Interacting with others (e.g. talking, shared activities, etc.).	“I love to chat with the taxi drivers, they know a lot, so I chat with the taxi drivers very often.” *(FGD*: *demographic details unavailable)*
26	Social relationships	The quality of the relationships a person has with other people or groups of people (e.g. close bonds, cohesiveness, harmony, love and support).	Having good relationships with family, friends and others.	“I mean you have to, you mix with your friends. I mean you go out with them, you know, like try to enjoy life a bit and then build up the relationship.” *(IDI*: *demographic details unavailable)* “So like we need, beside our family [sic], we need good friends. We also need family bond [sic]. In case when things don’t work out, we have a bond that we can…like a fate we can rely on.” *(FGD*: *49 years old*, *Chinese male*, *with medical condition/s 3 of the top 10 conditions and other conditions)*
27	Social role	A person’s identity in terms of what he does in relation to other people (e.g. takes care of friends and family) and his society (e.g. participation in social work, being active in national issues).	Having a role in the lives of others and being able to fulfil that role (e.g. being a good employee, a good wife, a good son).	“I think it’s good that, being a mother, you give of yourself; but you give until a stage where all your children are grown up.”*(FGD*: *56 years old*, *Chinese*, *female*, *with medical condition/s)* “There are certain social expectations, like when you get married- to have a house. Then I have a father who’s not working so I need to take care of him as well.”*(FGD*: *29 years old*, *Chinese*, *male*, *healthy)*

FGDP: Focus Group Discussion; IDI: In-depth Interview; HCW: Health Care Worker

Mental Health had the second largest number of domains, again spanning the various aspects of mental health. The domains of emotions and self-esteem covered the emotional aspects of mental health, while the domains of memory and active mind covered the more intellectual aspects of mental health. Interestingly, the largest number of domains (personal freedom, sense of growth, engagement with life, resilience and mindset) covered the more aspirational aspects of mental health.

Social Health was represented through three domains, covering the typically seen domains of social contact, social relationships and social roles.

Of note, the focus of 26 domains were on aspects of physical, mental or social health, which are related to usual functioning in healthy individuals, with only the domain of discomfort and pain being related to illness.

English-speaking participants generally gave a more subjective and broader view of health that encompassed physical, mental, emotional, social, spiritual, financial and environmental health. Finances and work were not cited as important aspects related to health or QoL. Rather, health was described as the basic necessity or requirements to engage in all other activities.

## Discussion

In this study, we developed a comprehensive, culturally sensitive conceptual framework [17] of Health domains in Singapore. We found three overarching areas of health—physical, mental and social—and identified domains in each area. To the best of our knowledge, this is one of a few such studies performed worldwide, and one of the first in an Asian sociocultural context.

The framework [5] we found in this study broadly parallels those found in the WHOQOL and PROMIS frameworks, with some notable exceptions. Our results are similar to and extend the findings of the WHOQOL and Patient Reported Outcomes Measurement Information System (PROMIS) teams, who observed health frameworks with physical, mental and social components. Our results differ from the framework of health used by the Short Form 36, which is a widely used HRQoL measure. The SF-36 framework [18, 19] of health used physical and mental components of health as a framework, with social health component incorporated into the mental health component. In contrast, in Singapore, the social and mental components of health are distinct. Our finding that the social and mental components of health are distinct helps to explain the results of previous studies of the SF-36 in Singapore, where in factor analysis, the social component of health did not load strongly onto the mental component of health. This same pattern was seen in several other Asian countries, suggesting that a similar three-component model of health may be present in these countries.

The domains of health identified in this study generally correspond to those identified in the WHOQoL [10] and PROMIS frameworks [19], with two exceptions. This correspondence was at the level of the physical, mental and social components of health and also at the level of specific domains within each component of health. Our results thus support and extend the findings of these previous landmark studies, and support the thesis that these three components of health are important across various socio-cultural contexts, including at least one such context in Asia (i.e. Singapore). Interestingly, the domains “Healing and Resistance to Illness” and “Bladder control” (both in the physical component of health), are domains not found on the WHOQoL or PROMIS frameworks (though they form part of several scales for example the Quality of Well Being Self-Administered Scale [20], the Sickness Impact Profile [21] and the Health Status Questionnaire). There are several possible reasons for this observation. First, these domains could be of greater importance in the context in which this study was conducted. The concept of “healing and resistance to illness” is common in Chinese culture [22], and could be related the concept of Yin and Yang [23]. A second possibility, which is less likely, is that a higher than expected proportion of participants had issues with bladder control—this would be unusual as these participants were largely drawn from the general population. Additional qualitative and quantitative studies need to be performed to further study these domains in the Singapore or Asian populations. Of note, we conceptualized financial and environmental factors, work and spirituality as factors that affect HRQoL, rather than being HRQOL domains themselves. During data analysis, we observed that text coded with these factors could map onto the HRQoL domains identified in the paper as modifiers of HRQoL. For example, spiritual factors mapped onto the domains of resilience and emotions. Another example is work—the quote “Work is important; it gives you a sense of purpose” shows that that it is not so much the work per se that is important for HRQoL but the fact that the individual derives a sense of purpose from the work.

We recognize several limitations of this study. First, while generalizability of the results to the Singapore population is reasonable (given that participants who were citizens or permanent residents with and without chronic illnesses), the generalizability to other urban Asian populations is unclear as their socio-cultural contexts may differ. Second, and participants who were purely Malay or Tamil speaking were not included in Focus Groups or Interviews because the impact of this on the results is likely to be small, given that pure Malay and Tamil speakers formed only 1.2% and 0.29% of the resident population in 2015, as the majority of Singapore residents are English-speaking [24]. Third, we recognize that there can be an element of subjectivity in categorizing these domains of health. As such, two coders independently analyzed each transcript to reduce this subjectivity. Overlapping of codes at times resulted in discrepancies between the two coders, which were resolved by a consensus process. As the objective of the study was to describe the framework of health domains in Singapore, we decided as a rule to retain all the health domains that were not sufficiently similar to be collapsed, to allow a rich qualitative description of this framework. An additional reason for this approach was to preserve the information that came directly from study participants through this pre-defined, rigorous coding process. We do recognize that these domains do need to be further evaluated using quantitative methods (e.g. factor analysis, various item response theory based approaches) and may be further refined through these approaches.

In conclusion, in this study, we found that the physical, mental and social components of health are present in the Singapore population, which is an urban Asian population of Chinese, Malays and Indians. These results provide a foundation for future research to rank the importance of domains of health in Singapore and in Asia. The results also form the basis for the development of item banks to assess HRQoL for important domains.

## Supporting information

S1 File S1 Moderator guide. (DOCX)
