# Supplementary material for: Developing a comprehensive, culturally sensitive conceptual framework of health domains in Singapore
Source: PLoS One. 2018 Jun 28;13(6):e0199881. doi: 10.1371/journal.pone.0199881 (PMC6023157; doi:10.1371/journal.pone.0199881)
Supplement: S1 File — (DOCX) [file pone.0199881.s001.docx]

## SHAWS Focus Group/Interview Facilitator Script

## INTRODUCTION & WELCOME (0 - 15 min)

General Introduction

Good morning to all, thanks for coming down…

To help me get you get more comfortable, let me introduce myself briefly… [facilitator introduces self]

Today, we’ve invited you to join our ***focus groups/interview*** to talk about **which areas of quality of life are important to you.**

Introduction to Focus groups (focus groups only)

May I know – has anyone joined a focus group before or know what it is about?

- Focus groups are a new and different way of doing research
- Focus groups are small group discussions that people talk about a topic
- Your views are the data that we are collect in research

Session Agenda (focus group only)

This is a group discussion , so I’ll be asking you questions and we’ll discuss these as a group, so it’ll be a pretty relaxed session.

[Refer to program schedule on the board]

Introduction to Research topic

The purpose of today’s discussion is to find out which areas of quality of life are important to you.

By “to you” we mean the local Singapore population. This is the first research study in Singapore to identify which areas of health are important to the QOL of local people.

We will discuss a few topics today. We will ask you what “quality of life” means to you, and to consider which areas are affected by health. We will also ask you what “health”, or being “healthy” means to you.

Administrative Points

So, before we relax into the discussion part, do allow me to get some administrative points out of the way…

1. In appreciation of you taking time to take part in this research study, we would now like to present you with a cash gift of $X.
   [Proceed to let participant sign acknowledgement form, pass token]
2. The interview will last approximately two hours including a break, but you may stop at any time during the focus group discussion if you no longer wish to continue. Quitting the focus group discussion will not change or jeopardize your medical care (for chronic patients) in anyway.
3. The focus group discussion/interview is being voice-recorded. However, it will be kept strictly confidential and it is solely intended for the researchers who are in charge of carrying out this survey. Due to the length of our discussion, it is necessary for us to record the discussion on digital voice recorder in order not to miss out important points. With the digital recordings, we do not need to return to you at a later date for any clarifications. Hence, this will be more convenient for you as well.
4. The questions that I am going to ask you are rather general. However, you are not obliged to answer all of them.
5. There is no right or wrong answers. Your responses will not be judged.

We would also like to find out more information about you, so could you fill in the background form labeled question set number 1?

[Respondents complete questionnaire Set 1]

Icebreaking (focus group only)

Alright, so now we’ve got the administrative part out of the way, let’s get to know each other a little better, as we’ll be discussing the questions as a group & it’ll be good if we’ll be more comfortable with each other. So we’ll go around each person for a short introduction. Do tell us your name or however you want us to address you (give labels) and something about yourself that you feel like sharing to the group. So for example, “I’m [facilitator name] and [something about self] ”

[Show them to write down name on name-cards]

At this point, don’t worry if you don’t know what to say, because we have two warm-up exercises, just to get everyone to brainstorm, and familiar with the topic that we are talking about.

So let’s just start with the warm-up exercises, to get you to think about these things.

1. **DISCUSSION ON QUALITY OF LIFE (45 min)**

[Purpose: To elicit the meaning of “quality of life”]

**Warm up exercise A**

| **Imagine** someone who is about your age (it could be you) and who is happy and satisfied with life as a whole.  How does his/her life look like?   - Can you write down the *important areas* of his/her life (e.g. family, work, social life, health, finances etc.), such that he/she is happy and satisfied with life? |
| --- |

- Could you please share with me/us, which areas of life did you write down?

[Note-taker to note on Chart/Whiteboard for focus groups]

- Are these the same areas of life what you consider to be important to you as well?
- What are the things which affect people’s quality of life?
  - [Probe] How does [specified area] affect your quality of life?
- Are there things which you have mentioned that you think are important to quality of life that people find it difficult to talk about?
- Are there things which we have not mentioned that you think are important to quality of life that people find it difficult to talk about?
- Are there any other areas of life that are important to you?

1. **DISCUSSION ON EXPERIENCES WITH HRQOL (45 min)**

We would like you to consider how your health is affecting, or has affected your quality of life. You can think of past experiences, or current experiences.

- - We would first like you to talk about your personal experiences, and personal viewpoints with health and quality of life.
  - However, we also understand that you may not have a personal experience, but have known someone very close to you who is sick, and think that that might be how your quality of life would be similarly affected.
- How has health/does health affect your quality of life?
- Can you give us some examples?
- What specific aspects make up this area? Can you describe this in further detail? (for example if you’ve mentioned “Physical health” it could comprise Energy level, Physical activity, Mobility, No Pain, etc.)
- Would you consider this aspect to be belonging to this specific area?
- Why are these important to you?
- *Besides these aspects, (broad area name) also includes the following aspects - *Refer to attached chart

**KIV**

- [ (If necessary) When we talk about “health” for the rest of the discussion , we are going to look only at specific areas of “health”.]
